# Patients’ and clinicians’ perceptions of oral anticoagulants in atrial fibrillation: a systematic narrative review and meta-analysis

**DOI:** 10.1186/s12875-021-01590-x

**Published:** 2021-12-22

**Authors:** Yeyenta Mina Osasu, Richard Cooper, Caroline Mitchell

**Affiliations:** 1 Academic Unit of Primary Medical Care, Faculty of Medicine, Dentistry and Health, University of Sheffield, Sheffield, S5 7AU UK; 2 ScHARR, University of Sheffield, Sheffield, S1 4DA UK

**Keywords:** Atrial fibrillation, Oral anticoagulants, DOACs, Warfarin, Patients, Elderly, Perceptions, Clinicians, Healthcare professionals, Systematic narrative review

## Abstract

**Background:**

Atrial fibrillation (AF) increases the risk of developing a stroke by 20%. AF related strokes are associated with greater morbidity. Historically, warfarin was the anticoagulant of choice for stroke prevention in patients with AF but lately patients are being switched or started on direct oral anticoagulants (DOACs). DOACs are promoted as safer alternatives to warfarin and it is expected that they will be associated with fewer challenges both for patients and healthcare professionals. This systematic narrative review aimed to explore perspectives of patients and professionals on medicines optimisation of oral anticoagulation with vitamin K antagonists and DOACs in atrial fibrillation.

**Methods:**

Prospero registration CRD42018091591. Systematic searches undertaken of research studies (qualitative and quantitative), published February 2018 to November 2020 from several databases (Web of Science, Scopus, Medline Via Ovid, CINHAL via Ebsco, and PubMED via NCBI) following PRISMA methodology. Data were organised using Covidence software. Two reviewers independently assessed the quality of the included studies and synthesized the findings (thematic analysis approach).

**Results:**

Thirty-four studies were included. Studies were critically appraised using established critical appraisal tools (Qualsyst) and a risk of bias was assigned. Clinicians considered old age and the associated complexities such as co-morbidities and the increased potential for bleeding as potential barriers to optimising anticoagulation. Whereas patients’ health and medication beliefs influenced adherence. Notably, structured patient support was important in enhancing safety and effective anticoagulation. For both patients and clinicians, confidence and experience of safe anticoagulation was influenced by the presence of co-morbidities,  poor knowledge and understanding of AF and the purpose of anticoagulation.

**Conclusion:**

Age, complex multimorbidity and polypharmacy influence prescribing, with DOACs being perceived to be safer than warfarin. This systematic narrative review suggests that interventions are needed to support patient self-management. There are residual anxieties associated with long term anticoagulation in the context of complexities.

**Trial registration:**

Not applicable.

**Supplementary Information:**

The online version contains supplementary material available at 10.1186/s12875-021-01590-x.

## Background

Atrial fibrillation is predominantly a condition of old age. The UK prevalence increases from 1.7% at age 60–64 years to 19.5% at age 85–89 years of age [1]. Older patients aged 65 years and over with AF are at greater risk of stroke and would benefit most from oral anticoagulation [2]. However, they are also at an increased risk of bleeding complications [3]. The benefits and risks are amplified in those who are 75 years old and over. Older patients with AF often have other co-morbidities resulting in concurrent use of multiple medications for long term conditions. Furthermore, other issues of complexity affect the care of the elderly, including frailty, propensity to falls, cognitive impairment such as dementia, and a higher incidence of acute and chronic renal impairment [3–5]. It is imperative therefore, that clinicians partner with older patients to provide regular monitoring and relevant information which is tailored to suit individual needs and circumstances. This will enable patients and encourage their active participation in long term disease management and self-care.

Patients should be provided with clear and unbiased information about the options between different OACs (vitamin K antagonists such as warfarin vs DOACs) and given time for reflection and questions. This is the basis of shared decision making and patient centred care. Patient preference, individual patient factors such as comorbidities and potential for drug interactions should be considered during the decision-making process [6]. NICE also recommends that alternative forms of anticoagulation such as direct oral anticoagulants (DOACs) be considered for people who are poorly controlled with vitamin k antagonists such as warfarin. Since 2012, there has been a steady upward trend in the use of DOACs for AF in the UK. Other parts of the world have also noticed a shift from vitamin k antagonists towards greater DOAC prescribing [7–9]. Despite the acclaimed benefits of DOACs, patient safety remains high priority for all healthcare professionals involved in DOAC prescribing, dispensing and monitoring. Increased gastro-intestinal bleeding is a significant adverse effect of DOACs, especially in patients treated with dabigatran, rivaroxaban and edoxaban, compared to warfarin. Although lower risk of fatalities associated with major bleeding have been attributed to DOACs compared to warfarin, many bleeding incidents have been reported in older adults and in those with poor renal function [10].

A previous qualitative systematic review concluded that physicians’ and patients’ perceptions and attitudes might be potential factors in the underuse of treatment with vitamin k antagonists [11]. However, more recent studies suggested that DOACs have resulted in an increase in the overall uptake of oral anticoagulant therapy [12]. The previous systematic review that examined clinician’s views and experiences of direct oral anticoagulants in the management of atrial fibrillation identified mostly quantitative, and only one qualitative study. Patient’s views were not represented, and the studies were only from Europe and U.S.A [13]. There is no systematic narrative review to date that has explored the quantitative and qualitative findings of patients’ and clinicians’ perceptions of medicine optimisation of oral anticoagulation (warfarin and DOACs). Medicines optimisation is a patient centred approach to ensure people get the right choice of medicines, at the right time, and are engaged in the processes by their clinical team [14]. Therefore, the objective of this narrative review is to identify perspectives of patients and clinicians on the optimisation of anticoagulation with vitamin K antagonists and DOACs in atrial fibrillation. To do so, we critically synthesised the qualitative and quantitative research evidence which explored patients’ and clinicians’ perceptions of safe and effective use of anticoagulants in older adults with atrial fibrillation.

## Methods

A review question was developed followed by a protocol and search strategy to ensure a systematic literature review process. A systematic narrative review was undertaken to synthesize the current evidence and literature relating to the perspectives on optimisation of anticoagulants in NVAF by patients and healthcare professionals. A protocol was developed and subsequently registered on PROSPERO (https://www.crd.york.ac.uk/prospero/ and registration number: CRD42018091591).

### Design

Systematic narrative review of qualitative and quantitative research using a pragmatic, integrated and narrative approach for synthesizing disparate evidence [15, 16].

### Data sources

Formal literature searches were carried out between 5th February 2018 and 25th May 2018 through several databases: Web of Science, Scopus, Medline Via Ovid, Cumulative Index to Nursing and Allied Health Literature (CINHAL via Ebsco), and PubMED via NCBI. The databases were searched from 1990 to 2018 and search terms were developed by all authors. The search strategy was based on specified characteristics from the review question using the SPIDER framework (Setting: primary care or secondary care, Population: older adults, Intervention: oral anticoagulants, Design: none specified, Evaluation: safety, effectiveness, adherence, prescribing or optimisation and Research type: Qualitative, quantitative). The database search included the use of the following Mesh (Medical Subject Headings) terms: gp OR practitioner OR “General Practitioner” OR physician* OR doctor* OR nurse* OR pharmacist* OR clinician AND aged OR elderly OR frail* OR “Old* adult” AND? oac OR anticoagula* OR apixaban OR dabigatran OR rivaroxaban OR edoxaban OR warfarin AND “non valvular atrial fibrillation” OR nvaf OR af. All citation identified on all databases were exported to a reference manager (Mendeley). Subsequently, all documents were imported into Covidence software platform to keep track of references, audit the selection process and to allow independent reviews by each reviewer. Duplicates were removed by the software at the import stage. Search alerts were set to notify the author of relevant publications after the formal review stage (beyond May 2018). This was done using the same search terms which was saved on the database but restricted to publication years 2018 to 2020. A repeat of the database search was conducted at a later date (26th November 2020) to check for any studies which may have been recently published or missed during earlier searches. This yielded six further studies which were then added to the final papers for review.

### Study selection

Three reviewers (YO, CM and RC) independently assessed the studies. Settings were created on Covidence to initially allow 2 reviewers (YO and CM) to screen titles and abstracts during the first stage. Review articles were excluded, and only original research was included in the full text reviews. However, the reference lists of the reviews were manually searched, and reference chaining was employed to obtain relevant studies for the next stage of the review.

The next stage involved reading and screening full texts of only original research based on the inclusion and exclusion criteria. Selected studies were extracted for inclusion in the review. Full text papers were read, and each supervisor read ten full text papers to verify that the literature matched the review criteria. Each study on Covidence was reviewed by YO and deemed either acceptable (assigned a ‘Yes’ vote); unacceptable (assigned a ‘No’ vote), or for consideration by the review team (assigned a ‘Maybe’ vote). Notes were made and attached to studies when needed as the review progressed. Documenting important notes and reasons for decisions taken, especially for those assigned ‘Maybe votes’, was helpful and served as an aide memoir for each reviewer during the face-to-face deliberations and discussions when resolving conflicting decisions. Where full texts were unavailable, the full text was requested from inter-library loans or corresponding authors were contacted directly by e-mail to request a copy of their transcript. All such requests were honoured. Unpublished research and grey literature were not included. Simple areas for clarification were resolved by discussion. Reference was made to the inclusion/ exclusion criteria at each stage of the review process, and by every member of the team to ensure a uniform standard. As stated earlier, CM was involved in the initial title and abstract screening and some full text screening, whilst RC was involved in the critical appraisal of included studies in the later stages of the review. All articles were screened against the inclusion and exclusion criteria stated in Table 1.

**Table 1:** showing inclusion and exclusion criteria of review papers

Inclusion criteria	Exclusion criteria
Studies published since 1995 (Global data)	Studies reporting on patient decision aids for oral anticoagulation
Studies published in English language	The prescribing, monitoring, adherence of oral anticoagulants for conditions other than non-valvular atrial fibrillation
Optimisation of oral anticoagulant medication. For example, a study may refer to aspects of medicines optimisation without specifically identifying this as such. Therefore, studies were included if they referred to safe prescribing, monitoring, adherence, safety, appropriate use, barriers to use, efficacy, adverse effects, or benefits of oral anticoagulants in elderly patients.	The prescribing, monitoring, adherence of oral anticoagulants for patients below 65 years of age
The attitudes, perception, views or experiences of healthcare professionals or elderly patients taking oral anticoagulants	Clinical trials of oral anticoagulants
	Studies reporting on prescribing trends or patterns of anticoagulants

### Critical appraisal and study quality

Due to the heterogenous nature of included papers we adapted the criteria for quantitative and qualitative studies from the QualSyst tool [17] onto the critical appraisal on Covidence platform. A custom risk of bias form was completed by each reviewer and for each included paper on the Covidence platform. Nevertheless, whilst some reviews do set a minimum threshold for inclusion based on a scoring system, the goal in this present review was to select studies of sufficient quality for inclusion. Therefore, studies were not graded as ‘high’, ‘medium’ or ‘low’ quality. Rather, a pragmatic, best-fit approach was adopted and studies of sufficient quality were included [16, 17].

### Data extraction and data synthesis

The data extraction form on Covidence was limiting due to the PICO (People (participants/population), Interventions, Comparisons and Outcomes) format for reporting and categorising studies as this was best suited to quantitative studies. Nevertheless, an extraction form was completed using the SPIDER (Setting, Population, Intervention, Design, Evaluation, Research type) framework in an excel spreadsheet [18]. This was used as a working document, made available to all three members of the review on a Google shared drive where more comments could be documented. A data extraction table was created where each research study was summarised. Supplementary Table 1 summarises each of the studies, including key findings and further comments. The integrated approach to synthesis [15] led to seven key thematic areas which are summarised in Table 2 and linked to the specific studies.

**Table 2:** A summary of identified themes from the literature

Theme 1	Theme 2	Theme 3	Theme 4	Theme 5	Theme 6	Theme 7
Medication safety concerns	Poor understanding	Older age	Co-morbidities	Practitioner/ patient confidence and experience	Patient support& adherence	Health & medication beliefs
Monette et al.*,* (1997) [19]	Lip et al (2002) [20]	McCrory et al.*,* (1995) [21]	Anderson, Fuller and Dudley (2007) [22]	Wang and Bajorek (2016) [23]	Al-Khalili, Lindstrom and Benson (2016) [24]	Alonson-Coello et al*,* 2015 [25]
Gross et al.*,* (2003) [26]	Rewiuk et al.*,* (2007) [27]	Monette et al.*,* (1997) [19]	Arts et al.*,* (2013) [28]	Yazdan-Ashoori et al (2017) [29]	Bastida et al.*,* (2017) [30]	Crivera et al.*,* (2016) [31]
Larock et al.*,* (2014) [32]	Frankel et al (2015) [33]	Granziera et al.*,* (2015) [34]	Armbuster et al.*,* (2014) [35]	Ikeda et al.*,* (2018) [36]	Ferguson et al.*,* (2017) [37]	Clarkesmith et al (2017) [38]
Alonso-Coello et al.*,* (2015) [39]	Glauser et al.*,* (2016) [40]	Basaran et al.*,* (2016) [41]	Rouaud et al.*,* (2015) [42]	Murphy, Kirby & Bradley (2020) [43]	Hanon et al.*,* (2016) [44]	Bartoli-Abdou, Patel, Xie et al (2018) [45]
Bajorek et al.*,* (2015) [46]	Wang and Bajorek (2016) [23]	Bertozzo et al.*,* (2015) [47]	Ferguson et al.*,* (2017) [37]	Bajorek et al (2007) [48]	Brown, Shewale and Talbert, (2017) [49]	Bartoli-Abdou, Patel, Crawshaw et al (2018) [50]
Crivera et al.*,* (2016) [31]	Clarkesmith et al (2017) [38]	Dantas et al.*,* (2004) [51]		Bartoli-Abdou, Patel, Crawshaw et al.*,* (2018) [45]		
Basaran et al*,* (2016) [41]	Dantas et al (2004) [51]			Bajorek et al.*,* (2009) [52]		
Clarkesmith, Lip and Lane (2017) [38]	Bajorek et al (2007) [48]					
McGrath et al.*,* (2017) [53]	Bajorek et al (2009) [52]					

## Results

**Fig. 1:**
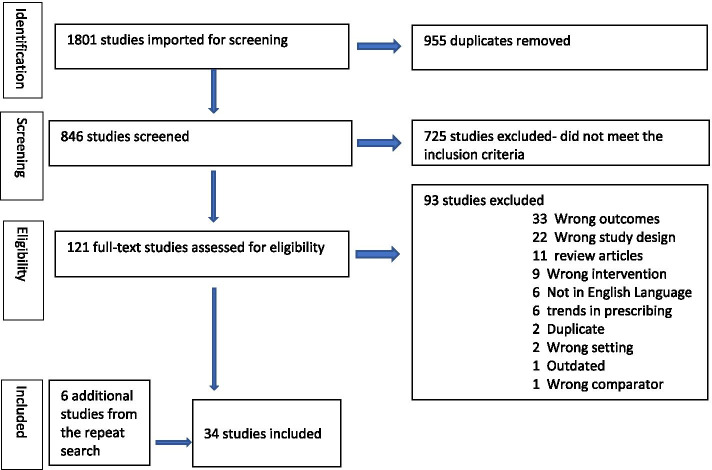
PRISMA Flowchart. The PRISMA diagram shows our search and selection process applied during the review

A total of 34 studies were included in this review all from OECD (Organization for Economic Cooperation and Development) countries. Eight were from U.S.A, five from United Kingdom and Australia respectively, three from Canada, two from Italy, Spain and France respectively and one from Poland, Netherlands, Belgium, Turkey, Sweden, Japan and Ireland respectively. Twenty-six of the studies were quantitative, six qualitative and two mixed method studies. These comprised 12 observational studies, 13 surveys, and one chart review. Six studies explored practitioner perspectives and 19 studies explored patient perspectives, and nine studies explored both. Sixteen of the studies included in this review are now over 25 years old and are pre- DOAC therefore, caution is advised when making deductions from the older studies. Overall, the quantitative studies were highly heterogenous in methodology, setting and inclusion criteria and type of oral anticoagulant. Twenty studies focused on warfarin, eight on DOACS and three on both. An additional word file shows this in more detail (Supplementary Table 1). The PRISMA diagram in Fig. 1 represents the data extraction process.

### Thematic analysis

Seven main themes were identified and are summarised in Table 2 where the studies are grouped by themes. The themes reflected a range of different aspects of the safe and effective use of oral anticoagulants centred around clinically orientated aspects of the patients such as co-morbidities and older age, but also more subjective aspects such as perceived safety concerns, confidence and experience of prescribing doctors and also knowledge and support.

### Medication safety concerns

The most frequently identified theme related to physician concerns, uncertainty and anxiety about causing bleeding related harm. Specifically, these harms were mainly associated with bleeding, especially in patients with a tendency for falls, suggesting that physicians were averse to causing bleeding related harm [19, 26]. The cautious attitude of prescribers seems to have persisted. For example, a Spanish study reported that patients were more willing than physicians to accept a high frequency of bleeds with warfarin over a two-year period to avert a stroke [39]. These concerns did not appear to fade with the introduction of DOACs as two studies reported doctors under-prescribing DOACs for fear of causing bleeds [32, 41]. Furthermore, the complexities and burden of therapy in elderly patients who often have co-morbidities as well as impaired cognitive and functional ability further contribute to concerns about medication safety [31, 45, 46].

### Poor understanding

Themes around poor understanding of atrial fibrillation and anticoagulation were identified in nine empirical studies, particularly in relation to patients. Despite a long duration of known AF and anticoagulation, patients were unable to recall their actual heart condition and displayed poor understanding of the rationale for their treatment regardless of whether they were on warfarin or a DOAC [20, 27, 38, 52]. Apart from patients, healthcare professionals sometimes also displayed poor understanding of anticoagulation management and treatment [33, 40, 48]. Although physicians claimed that they were likely to initiate discussion about stroke in AF related stroke, patients in one study expressed dissatisfaction with the quality of education and information they received from physicians [33]. Sometimes, doctors did not follow any specific guideline or scoring tool, rather they made recommendations based on their own personal preference, clinical judgement and experience [23, 40], thus highlighting areas of educational need for clinicians and barriers to anticoagulation in the management of AF.

### Older age

The impact of old age on anticoagulation was evident across the literature but was more prominent in six studies. For example, although physicians understood the need for anticoagulant treatment for stroke prevention, they remained reluctant to prescribe anticoagulants for older patients. Particularly, those aged over 75 were managed most conservatively, and in situations when they were prescribed anticoagulants, this was done in lower intensity because prescribers believed that anticoagulation was more complex in the older patient group [19, 21].

### Co-morbidities

Co-morbidities presented an extra layer of complexity and uncertainty to decision making when considering anticoagulation in elderly patients. For example, doctors were reported to be risk averse and reluctant to prescribe warfarin for patients with AF and a history of falls in a Canadian study [19]. In other scenarios, doctors showed a wide range of responses which were attributed to uncertainty about risk and benefit. Doctors also preferred sharing the decisions and responsibility of prescribing with the patient especially for complex cases, and seeking further risk information from specialists and this often led to inappropriate prescribing decisions [28, 54]. Further, co-morbidities and factors relating to old age including cognitive dysfunction, frailty and the fear of falls have also been associated with poor anticoagulation control and adherence [37, 42].

### Practitioner/ patient confidence and experience

The level of familiarity a prescriber had with anticoagulant therapy and experience of use in clinical practice helped improve confidence and reduced some uncertainties associated with anticoagulants. Although DOACs have increasingly become diffused in primary care a qualitative Australian study reported that most healthcare professionals preferred prescribing warfarin due to their unfamiliarity with DOACs [23]. Similarly, lack of clinician experience with DOACs was reported in another study [29]. Contrary to this, a more recent U.K study found that patients had low risk perception and an overwhelming preference for DOACs over warfarin [50].

### Patient support and adherence

From the patients’ perspectives, various forms of structured support from healthcare professionals, friends, carers or family were important for the successful optimisation of anticoagulant therapy. Patient education using motivational interviewing, structured patient support and follow up system greatly improved adherence in both users of apixaban and rivaroxaban in one Swedish study [24]. Similarly, an integrative approach to patient support provided by community pharmacists through close monitoring and validation of prescription was reported to improve appropriateness of anticoagulation in primary care [30]. Not surprisingly, adherence improved when elderly patients developed a routine and had family around to support them with medicines taking [55].

### Health & medication beliefs

Finally, the impact of patient’s beliefs on adherence was identified in some studies and this influenced decisions about treatment such as adherence and necessity of medication [31, 38]. Two studies highlighted issues surrounding patients’ misconception of atrial fibrillation and poor understanding of the aims of anticoagulant treatment. Although patients with AF had more co-morbidities, they were less likely to recognise the burden of AF as it was just one of their many illnesses. Consequently, such patients did not always recognise the necessity of their anticoagulant therapy to prevent a stroke [45, 50]. However, patients regarded the authority and expertise of healthcare professionals (physicians and pharmacists) highly and were more likely to adhere to medication choice or decisions based on the doctor’s recommendations as they believe “the doctor knows best”. Although the perceptions and attitudes of patients vary and are influenced by different factors, patients’ beliefs especially when influenced by a health professional may encourage willingness to comply with the doctors wishes.

## Discussion

### Main findings

**Fig. 2:**
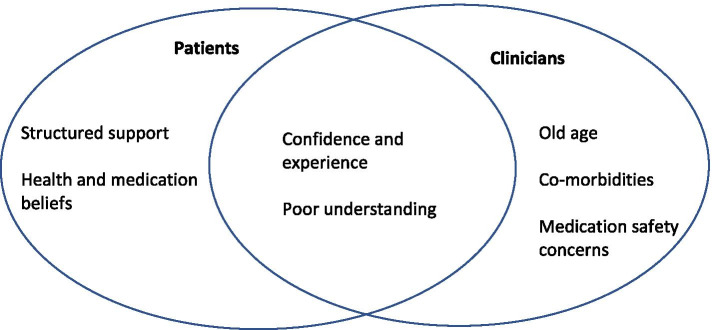
Identified themes.The figure shows themes that were predominantly identified in patients’ and clinicians’ perspectives and themes that were common to both patients and clinicians

In this systematic narrative review exploring the perceptions of oral anticoagulants, several themes were identified (Fig. 2) which could explain the factors that underpin the attitudes and views of patients and clinicians. Clinicians considered old age and the associated complexities such as co-morbidities and the increased potential for bleeding as potential barriers to optimising anticoagulation. Whereas, patients’ health and medication beliefs influenced adherence, it was also noted that structured patient support was important in enhancing safety and effective anticoagulation. For both patients and clinicians, confidence and experience of safe anticoagulation was influenced by the presence of co-morbidities, poor knowledge and understanding of AF, and the purpose of anticoagulation.

### Findings in the context of previous research

Two themes- practitioner/ patient confidence and experience, and poor understanding were common to patients and clinicians. Several studies reported clinician preference for warfarin over DOACs due to lack of sufficient experience with the latter. Most of these studies were carried out with warfarin or in the early years when DOACs were relatively new [23, 26, 29, 43, 48]. A recent meta-synthesis characterized clinician’s beliefs and experiences of oral anticoagulation in AF [56]. However, in addition to clinician’s views, this present research includes mixed methods research, explores patients’ views and experiences. Particularly, this present review highlights the importance of patients’ health and medication beliefs on treatment decisions, choice and adherence.

A recent research suggest that physicians are becoming less risk averse and more keen to prescribe anticoagulation for stroke prevention [36]. Moreover, other studies report increasing patient confidence and experience. Notably, there is an overwhelming preference for DOACs over warfarin amongst patients [50]. Patients and healthcare professionals alike showed poor understanding of anticoagulation management and treatment. However this was more prominent with patients [20, 27, 52]. This highlights the need for better information that is targeted to the patient and clinician to aid consultations and shared decision making. Ongoing support and education to both patients and clinicians is important for best practice and adherence [57]. Further findings from a qualitative study by Borg et al., highlighted the need for patients and doctors to adopt a new model of medical consultations which improves the engagement and active participation of both parties in decision making [58]. Moreover, other studies have suggested that patient and clinician education alone is not sufficient. Additional measures such as providing regular support, re-enforcing information and behaviour change techniques are important strategies to enhance the optimisation of anticoagulants when incorporated with information provision and patient education [11, 48, 59]. It is likely however, that doctors’ attitudes and perceptions about the adverse effects of anticoagulation in the elderly is changing with the innovation of DOACs. The normalisation of these newer agents in routine practice may be responsible for the changing attitudes. The study by Bajorek et al., (2015) suggests that the introduction of direct oral anticoagulants may have shifted doctors’ focus on bleeding risks and monitoring towards more practical aspects of anticoagulant.

Instead of being overly cautious and concerned about bleeding doctors are now giving careful consideration for complexities such as adherence, impaired cognitive and functional ability of the patient during the decision making process [46]. The findings from this review show that poor patient and practitioner knowledge, older age, co-morbidities, history or fear of falls and bleeding all act as barriers to safe and effective anticoagulant optimisation. However, structured educational support facilitates safe use. These findings can be traced to literature about patient centred care, patient safety, shared decision making and lay knowledge [60].

There were also some conflicting reports within the studies included in this systematic narrative review. For example older age and co-morbidities were considered barriers to effective anticoagulation in some studies [27, 34, 37, 53, 61]. However, another study reported that patients with a higher CHA2DS2-VASc score, prior bleeding and higher morbidity were more adherent to their anticoagulant medication [38, 62]. There may be the perception among physicians that lack of routine monitoring with DOACs may lead to poor medication adherence, but it is possible that older people living with AF and other long term conditions may have heightened perception and sensitivity for the necessity of medication due to maintaining regular contact with the healthcare system as a result of co-morbidities and polypharmacy. Therefore, the daily routine and patient work to manage long term conditions may act as a prompt for patients to take their anticoagulant medication in line with their other daily medication routine.

When considered in the context of guideline-adherent oral anticoagulant prescribing, this review highlights the importance of sustained healthcare professional support to improve guideline adherent prescribing. It is also necessary to consider patients’ health and medication beliefs to facilitate shared decision making and improve adherence of oral anticoagulants. The impact of patient beliefs about prescribed medication among older patients with polypharmacy was explored recently. The authors found that patients displayed a mixture of positive and negative attitudes towards medication, and this may be influenced by the doctor- patient relationship [63].

Some studies highlighted issues surrounding patients’ misconception of atrial fibrillation and poor understanding of the aims of anticoagulant treatment. It is evident from the review that there is a strong direct relationship between patient knowledge and the quality of anticoagulation. Therefore, structured patient and healthcare professional education and support is crucial for optimised anticoagulation to prevent stroke in at risk patients whilst maintaining patient safety and practitioner confidence.

### Limitations and strengths

This is the first large systematic narrative review which explores patient and professional perspectives on the safe and effective of anticoagulants which includes both quantitative and qualitative research. The strengths of this review include the development of a well-defined review question with set inclusion and exclusion criteria which was agreed by all members of the review team. Therefore, all abstracts, titles and full texts were judged based on this criterion. Covidence was a useful tool for organising, storing and keeping track of team progress. Though Mendeley was used as a reference manager during the course of this review, functions within covidence meant that each reviewer could see how much work was done and what was required of other team members as per team settings. Additionally, functions such as automatically creating a PRISMA diagram as the review progressed made the process more transparent. The individual log-in meant that each reviewer could only see their own work, and not those of other team members reducing the risk of selection bias until consensus meetings to discuss conflicts. Furthermore, the review tool kept an audit trail of who did what, and why. However, as stated earlier, covidence was limited in the overtly quantitative format and use of PICO in the data extraction forms, hence an alternative format was developed on google drive. Nevertheless, there was some scope to customise the form to fit with certain aspects of the review as necessary. Finally, only original research from published literature was included in this review. Grey literature (unpublished work, and work from non-academic journals) were not included in this review. There is therefore a risk of introducing publication bias. As stated earlier, 16 of the included studies are over 25 years old therefore, it is therefore likely that practice and perceptions towards anticoagulants have changed over time. The present synthesis may be limited in summarizing similarities and differences in the views of patients and clinicians. Therefore and area for future work could be further assessment of findings, for example, taking into account naïve vs experienced/ switched treatment status, prior stroke, patients’ socio-demographic, anticoagulant affordability and care setting.

## Conclusions

AF is a chronic disease which can increase the risk of stroke in older adults especially in the context of co-morbidity. This is important because AF associated strokes are linked to greater morbidity. Oral anticoagulants are viewed as effective medication for stroke prevention in patients with non- valvular atrial fibrillation. However, concerns over advancing age, co-morbidities and adverse bleeding events has ramifications for their optimisation, especially in the elderly. Findings of this systematic narrative review provide some evidence for the need to support both older patients and clinicians to reduce the residual anxieties associated with long term anticoagulation in the context of complexities. Consequently, understanding and confidence may be improved by providing structured educational support to healthcare professionals and patients.

## Supplementary Information

**Additional file 1: Supplementary Table 1.** Extraction table showing summary of studies**.** This is a tabular representation of all original research included in the systematic narrative review.

## Data Availability

The dataset(s) supporting the conclusions of this article is (are) included within the article (and its additional file).

## Abbreviations

AF: Atrial fibrillation
DOACs: Direct oral anticoagulants
CHA_2_DS_2_VASC score: Congestive heart failure (1), Hypertension (1), Age ≥ 75 yrs. (2), Diabetes (1), Stroke or transient ischaemic attack (2), Vascular disease (1), Age ≤ 65 yrs. (1), Sex category (1)
HASBLED Score: Hypertension (1), Abnormal renal/ liver function (1), Stroke (1), Bleeding history or predisposition (1), Labile INR (1), Elderly (1), Drugs/alcohol concomitantly (1**)**
ATRIA: Anticoagulation and Risk Factors in Atrial Fibrillation score
PICO: Population, Intervention, Comparisons, Outcomes
