# Supplementary material for: Patients’ and clinicians’ perceptions of oral anticoagulants in atrial fibrillation: a systematic narrative review and meta-analysis
Source: BMC Fam Pract. 2021 Dec 22;22:254. doi: 10.1186/s12875-021-01590-x (PMC8697449; doi:10.1186/s12875-021-01590-x)
Supplement: Supplementary file 1 — Additional file 1: Supplementary Table 1. Extraction table showing summary of studies. This is a tabular representation of all original research included in the systematic narrative review. [file 12875_2021_1590_MOESM1_ESM.docx]

**Patients’ and Clinicians’ Perceptions of Oral Anticoagulants in Atrial Fibrillation:**

**A systematic narrative review and meta-analysis**

Yeyenta Mina Osasu MPharm PGDipClinPharm*

[yeyenta.osasu@nhs.net](mailto:yeyenta.osasu@nhs.net)

Academic Unit of Primary Medical Care

Faculty of Medicine, Dentistry and Health
University of Sheffield

S5 7AU

Dr Richard Cooper BPharm MA PhD PGCertHEd

[richard.cooper@sheffield.ac.uk](mailto:richard.cooper@sheffield.ac.uk)

Senior Lecturer in Public Health
ScHARR, University of Sheffield
Sheffield, S1 4DA, UK
+44 (0)114 2220768

Dr Caroline Mitchell MD FRCGP MRCGP PGCertMedED

[c.mitchell@sheffield.ac.uk](mailto:c.mitchell@sheffield.ac.uk)

Academic Unit of Primary Medical Care

Faculty of Medicine, Dentistry and Health
University of Sheffield

S5 7AU

Yeyenta Osasu*- 1^st^ author/ Corresponding author

**Supplementary Table 1** Extraction table showing summary of studies

| **Author** | **Methodology** | **Participants/ Country** | **Objectives** | **Main findings** | **Limitations/ Comments** |
| --- | --- | --- | --- | --- | --- |
| McCrory *et al.* 1999 (17) | Quantitative | 450 Physicians  U.S.A | To determine the influence of age on the prescribing of anticoagulants by physicians | Physicians were less likely to prescribe anticoagulation for 75-year olds than younger patients aged 55-65 years. Lower intensity anticoagulation was preferred in older patients especially when aged over 75 years | The low response rate of physicians could introduce a selection bias. |
| Monette *et al.,* 1997 (18) | Quantitative | 182 Physicians in primary care  Canada | ﻿To assess the knowledge and attitudes of physicians regarding the use of warfarin for stroke prevention in patients with atrial fibrillation in long-term care facilities. | Physicians were more averse to bleeding than they were to stroke and were reluctant to prescribe warfarin due to patient’s fall risk, especially in older patients ≥75 years. | This is a very dated study. The stroke and bleeding risks were based on arbitrary measures and not recognised schemas such as HASBLED, CHA_2_DS_2_VASC or ATRIA for example. |
| Lip *et al.,*2002 (11) | Quantitative | 119 patients  U.K | To assess if there were differences in perception to AF and anticoagulation amongst different ethnic groups | Poor perception of AF and warfarin amongst ethnic groups regardless of duration of therapy. Only a minority of black and minority ethnic felt they had enough information about their warfarin. A significant proportion of patients felt their heart condition was not serious. | This study is dated but highlights the need for improved patient education from healthcare professionals especially in those from minority ethnic groups. There is a gap in what the patient actually knows and what the doctor thinks he or she knows. |
| Gross *et al.,* 2003 (20) | Quantitative | 142 General internists  U.S.A | To identify physician’s attitudes and beliefs associated with warfarin use | The prescription of warfarin for NVAF was not driven by perceived benefits of anticoagulation. Rather physicians were more risk averse. The fear of causing intracerebral haemorrhage made them less likely to prescribe warfarin. | Case vignettes were used in this study. Perhaps physician’s decision would be different if they had real patients. Also response bias (low response rate) was a limitation to this study. Very dated study. |
| Dantas *et al.,* 2004 (21) | Qualitative | 21 Patients  Canada | To understand the experience and perspective of individuals on warfarin for atrial fibrillation | Practitioners were paternalistic in decision making and patients had high trust in the doctor’s decisions. Poor patient understanding of treatment and low retention of benefits and risks of therapy, especially in the elderly (≥75 yrs). However, there was high satisfaction and low impact on daily living. | Patients reported only minor inconveniences and saw warfarin as just another daily pill. |
| Rewiuk *et al.*,2007 (22) | Quantitative | 61 patients  Poland | To assess patients’ knowledge of anticoagulation and the effect on INR control | Low patient knowledge of antithrombotic prophylaxis amongst patients with AF. There was a strong correlation between patient knowledge INR control. Age and was inversely correlated with INR control | This study demonstrates the importance of patient education and adequate anticoagulation.  Limitations include low sample size, hospital recruitment risks selection bias of sicker patients with potentially lower knowledge of their medication. |
| Anderson, Fuller and Dudley, 2007 (23) | Mixed methods | ﻿14 Senior physicians  United Kingdom | ﻿To understand physicians’ behaviours and attitudes toward anticoagulation for AF, examine why risks are misperceived and how clinicians cope with risk and benefit dilemmas | Wide variation in the decision to anticoagulate. Physicians remained uncertain and concerned about knowledge of risk and benefit. | The use of vignettes limits the information available to physicians in a real-life situation when making treatment decisions. Pre-dates DOACs, comparing warfarin vs. aspirin for stroke prevention |
| Bajorek *et al*.,2007 (24) | Qualitative | 63 patients and health professionals  Australia | To explore the views and attitudes of healthcare professionals, patients and carers on warfarin management | All participants groups reported a lack of information and targeted patient communication to support decisions and ongoing management. | Good range of participants encompassing hospital doctors, GPs, nurses, patients and carers |
| Bajorek *et al*., 2009 (25) | Qualitative | 17 patients and carers  Australia | To explore the perspectives of elderly patients and/ or their carers regarding the use of warfarin therapy | Participants expressed having poor understanding and information regarding warfarin therapy. Anxiety and complexities of warfarin therapy were also a concern. | Elderly patients tend to be more accepting of warfarin therapy in contrast to the perceptions of health care professionals. |
| Arts *et al.,* 2013 (26) | Quantitative | 981 patients  36 general practitioners  Netherlands | ﻿To determine adequacy of antithrombotic treatment in patients with non-valvular atrial fibrillation. To determine risk factors for under- and over-treatment. Design: | ﻿The majority (59%) had a CHADS2 score ≥2 and 69% were treated with OAC . More than 16% of all included patients had one or more contraindications for OAC use. One or more comorbidities were present in 81% of patients, with hypertension being the most prevalent (57%) . | The study was done in 2008 when CHADS_2_ was used to assess stroke risk. It is likely that more patients may have been classified as undertreated if the current CHAD_2_VAS_2_C stroke risk calculator was used in assessment.  Retrospective nature of the study introduces bias and difficulty in assessing the rationale behind the clinical judgement at the time |
| Armbruster *et al.*, 2014 (27)  ﻿ | ﻿Quantitative | 458 Patients  U.S.A | ﻿To evaluate the use of dabigatran at a community hospital and identify prescribing areas that can be improved to ensure appropriate use and patient outcomes | 14% of participants were excluded due to incomplete documentation and inability to assess renal function. Of the included patients, 16.6% were treated with inappropriate regimens including receiving too high a dose based on renal function, once daily dosing, concomitantly taking an interacting drug and continuing with an inappropriate regimen at home. | This retrospective chart review only focused on the use of dabigatran at a single community hospital. The retrospective nature could introduce bias as patients were not being directly observed resulting in heavy dependence on documentation by nurses and doctors. |
| Larock *et al.*, 2014 (28) | Quantitative | 69 patients  Belgium | To prospectively evaluate the appropriate prescribing of dabigatran and rivaroxaban in patients with NVAF | Inappropriate prescribing of DOACs was recorded in about half of the patients, and about the same number experience adverse events. Poor compliance in 16% of patients. | This study was done in 2013 when rivaroxaban and dabigatran were still novel, this may explain the inappropriate prescribing. The normalisation of DOACs in routine practice may have improved practitioner confidence and prescribing. |
| Alonso-Coello *et al.,* 2015 (29) | Quantitative | 96 patients  96 physicians  Spain | To characterize the ﻿distribution of patient and physician values and preferences in the decision making in starting anticoagulation prophylaxis | ﻿Wide variability in patient and physician values and preferences regarding the trade-off between strokes and bleeds. Patients were more stroke averse than physicians | 86% of the patient participants had only elementary or no formal education and this study included patients who were at risk of developing AF, rather than patients who actually had the condition. |
| Frankel *et al.,* 2015 (30) | Quantitative | 499 patients  203 Carers  507 Doctors  U.S.A | To understand perspectives on effective communication and barriers to optimal anticoagulation | Conflicting perspectives between physicians and patients. Improved communication and education is needed to optimise management of AF and stroke prevention | Highlights the educational needs of patients and included a variety of stakeholders. The survey instrument limits the descriptions, explanations and nuances which can be explored through qualitative research. |
| Rouaud *et al.,* 2015 (31) | Quantitative | 2164 patients  France | To assess the impact of co-morbidities on INR control | Co-morbidities, haemorrhagic events and hospitalization were associated with poor INR control and quality of anticoagulation | Patients in this study were aged 80 years and over. This may introduce selection bias. Further research with patients aged 65-79 with co-morbidities will help reduce confounding factors. |
| Bajorek *et al.,* 2015 (32) | Quantitative | 50 general practitioners  Australia | ﻿To survey GPs regarding their approach to managing AF, particularly in relation to stroke prevention therapy, and to identify the range of services to support patient care.  Methods | When making decisions to initiate anticoagulant therapy for stroke prevention, GPs considered the stroke risk, adherence and management. The perceived benefit of therapy, associated risks and issues about medication safety. | The findings of this study are based on the management of AF patients where warfarin has been the mainstay but the approach to management, monitoring and decision making are likely to evolve with DOACs. |
| Granziera *et al.,* 2015 (33) | Quantitative | 798 patients  Italy | To retrospectively assess the incidence of major bleeding in NVAF patients who are over 80years old | Very elderly patients aged over 85 years were at higher risk of suffering major and fatal bleeding events than patients who were 80-84 years of age. | Retrospective cohort stud and patients were on warfarin. Patient and practitioner views were not sought. There is also an association between age and bleeding in elderly patients with DOACs |
| Bertozzo *et al.,* 2016 (34) | Quantitative | ﻿798 patients  Italy | ﻿To analyse the reasons for warfarin discontinuation in a real-world population | Patients with vascular disease, age ≥80years,labile INR and those with increased bleeding risk were more likely to discontinue treatment. Ninety-two percent of decision to discontinue were made by made by healthcare professionals- main reasons were low life expectancy, frailty and bleeding side effects. Other reasons were monitoring difficulties and poor compliance | This study shows that anticoagulation in the very elderly still presents a challenge. This study was based on warfarin alone. Further research is needed to explore if the introduction of DOACs improve the quality, and thus the persistence of anticoagulant treatment to achieve improved patient outcomes. |
| Glauser *et al*., 2016 (35) | Quantitative | 51 Cardiologists 50 primary care physicians  U.S.A | To determine the attitudes and practice patterns of doctors regarding anticoagulation in AF. | Cardiologists relied on Stroke risk scores for clinical assessment whereas primary care physicians relied on clinical judgement and experience. | This study was performed in spring 2012 before guidelines were updated so attitudes and prescribing behaviour patterns of clinicians is likely to have since changed. DOACs still very new. |
| Hanon *et al.,* 2016 (36) | Quantitative | 51 cardiologists and 422 patients  France | To assess patient satisfaction after switch from VKA to rivaroxaban | Improved patient satisfaction and adherence with rivaroxaban, assessed by burden and benefit scores. Patients over the age of 75 had a higher discontinuation rate mainly due to adverse bleeding events. | A large majority of the physicians in this study were from private practice and this could influence the type of patients and responses. More than half of the patients in this study were not prescribed the appropriate dose of rivaroxaban based on eGFR. |
| Wang and Bajorek, 2016 (37) | Qualitative | 26 healthcare professionals  7 specialist clinicians  6 GPs  7 Pharmacists  6 Nurses  Australia | To explore healthcare professionals’ views on the use of oral anticoagulants for stroke prevention in AF | Decision making is based on the doctor’s preference and influenced by the specialty and the types of patients they see. Pharmacists and nurses were more concerned with issues pertaining to patient safety and medication management. | This study was carried out in 2014 when DOACs were still novel and there was some unfamiliarity with their use, Attitudes may have since changed as standardised guidelines and new evidence emerge. |
| Crivera *et al.,* 2016 (38) | Quantitative | 1184 patients  U.S.A | To assess patients’ reasons for nontreatment and their attitude towards anticoagulant treatment and dosing regimens | Bleeding concern (followed by medical need) was the major reason for stopping anticoagulation, even though patients were classified as having a high stroke and low bleeding risk. Fifty seven percent of patients who were never anticoagulated were on aspirin for AF suggesting an underestimation of stroke risk. Patients reported taking a pill once a day more favourable. | Potential sources of bias for this paper include: Recruitment and sampling for this study were not stratified. ATRIA scores were used to assess bleeding risk rather than HASBLED. The authors are employees of Janssen, which is the marketer for rivaroxaban in U.S.A.  The difference in patient preference between taking a pill once Vs. twice a day may not be statistically significant but this was only reported using descriptive statistics of mean and standard deviation |
| Basaran *et al.*, 2016 (39) | Quantitative | 2086 patients  Turkey | To investigate the potential misuse of NOACs and effect of adherence to recommendations | Older patients with good renal function and low bleeding risk were more likely to be undertreated with dabigatran whilst patients with impaired renal function, and increased risk of bleeding were more likely to be overtreated with rivaroxaban | This was a subgroup from another larger study. It is possible that the study design may introduce some bias in its reporting. Cross sectional study is a snapshot and does not explain the reasons for overtreatment or undertreatment |
| Al-Khalili, Lindstrom and Benson, 2016 (40) | Quantitative | 301 patients  Sweden | ﻿To assess and compare adherence to rivaroxaban and apixaban inpatients with non-valvular AF | ﻿High estimated adherence levels for both  apixaban and rivaroxaban when initiated in a well-structured atrial fibrillation clinic and no statistically significant difference in persistence during one-year treatment time.  Patient education using motivational interviewing and proper patient selection, structured patient support and follow up system were cited as reasons for the high level of adherence. | Retrospective study, method of assessing adherence was unreliable and could introduce bias and short follow up time.  Though the study reports high adherence levels, this study was done in a highly structured clinical setting which required patients to attend appointments for follow up. Also, general adherence for both NOACs reduced over time. |
| Bastida *et al.,* 2017 (41) | Quantitative  Primary Care | 101 patients with AF  Spain | To describe the use of DOACs and analyse their prescribing in primary care. To evaluate possible factors associated with adverse events and the usability of prescription support forms. | Most patients were on rivaroxaban or dabigatran. Increased bleeding risk in 47% due to concomitant medication or illness. 90% of patients were receiving appropriate doses. Thirty six percent of patients had a high HASBLED score of 3 or more. Ten patients reported adverse events- 3 cases of haematuria with dabigatran and 3 cases of 3 cases of rectorrhagia with rivaroxaban | Unlike the findings from Basaran, et al 2015, here, 90% of patients received appropriate doses. This may be due to the close monitoring and validation of prescription forms by community pharmacists.  This study re-emphasises the need for closer follow up and monitoring of adverse events, especially in elderly patients. |
| McGrath *et al.,* 2017 (42) | Quantitative | 1,405 patients  U.S.A | To identify barriers to anticoagulation in older adults at high stroke risk | Despite high stroke risk scores, 44% of participants were discharged without oral anticoagulation. Strong associations between older age, dementia, history of major bleeding, and disability and omitted oral anticoagulation at discharge. | Anticoagulation may have been withheld for accurately assessed poor prognosis resulting in early mortality post discharge. This study was based on attitudes of physicians in 2003 to 2009, this could have changed with time. |
| Clarkesmith, Lip and Lane, 2017 (43) | Qualitative | 16 patients  U.K | ﻿To explore patients’ experiences, and perceptions of AF and anticoagulation, and the educational needs of AF patients taking dabigatran for stroke prevention | Varied patient experiences with some contradictions in themes. The main themes: patients’ understanding of diagnosis, reaching a treatment decision, the challenges of living with OAC, perception of treatment, challenges of living with AF and patient recommendations | This was a single centre study based on only one NOAC- dabigatran. Therefore the generalisability of the findings may be limited. |
| Yazdan-Ashoori *et al.,* 2017 (44) | Quantitative | 247 resident doctors  Canada | To assess the choices of trainee doctors when choosing anticoagulation for patients with atrial fibrillation | Doctors preferred warfarin over DOACs in uncomplicated cases but chose NOACs if there was a history of labile INR. | Resident doctors are still in training thus their attitudes and responses suggest that further training is required in this area for them. Self-reported nature of the study means the results may not be generalisable. |
| Ferguson *et al.*,2017 (45) | Qualitative | 144 patients  Australia | To identify the barriers and facilitators to anticoagulation in patients with CHF and concomitant AF from the perspective of patients and providers | Barriers to anticoagulation include age related factors e.g cognitive impairment, frailty and falls. People who had a routine or family support were more adherent with treatment | The majority of quotes were from informal bedside interviews, and they were not recorded or transcribed verbatim. However, there was good reflexivity by the researcher. Warfarin was the anticoagulant in this case. |
| Brown, Shewale and Talbert, 2017 (46) | Quantitative | 15,348 patients with AF  U.S.A | ﻿To compare adherence and treatment patterns among NOACs for stroke prevention in patients with nonvalvular atrial fibrillation (NVAF) | Patients on dabigatran had lower adherence and were more likely to change to another anticoagulant older patients and those with a higher stroke risk were more likely to have higher rates of adherence | Data for this study was based on a large claims database using the proportion of days covered. Assumptions are being made here that patients actually consumed their medication and were compliant with the dosing regimen but this cannot be confirmed and ﻿in practice this does not give the whole picture regarding medication adherence. |
| Bartoli-Abdou, Patel, Xie *et al.,* 2018 (47) | Quantitative | 311 Patients  U.K | To investigate differences in the beliefs between those with optimal and suboptimal anticoagulation control | Patients taking anticoagulants for AF experienced less treatment burden and were less likely to recognise the necessity and benefits of therapy compared to venous thrombolytic patients. | This study about illness and medication beliefs also included patients taking anticoagulants for venous thromboembolism. |
| Ikeda *et al.,* 2018 (48) | Quantitative | 313 patients  38 physicians  Japan | To investigate reasons for warfarin continuation in the despite the availability of DOACs | Physicians considered DOACs safer and more effective than warfarin but about 50% of patients were not counselled to switch because of stable INR and reduced renal function. Reasons for patient continuation were stability of warfarin treatment and INR, familiarity with, and low cost of warfarin. | This study design and methodology may lead to bias as the authors disclosed that the editing of the manuscript was supported by pharma companies who market DOACs. |
| Bartoli-Abdou, Patel, Crawshaw, *et al.*,2018 (49) | Qualitative | 20 Patients  U.K | To understand the real work experiences of patients switched from warfarin to DOACs | Patients considered warfarin to be the same as their other medicines however the inconvenience of therapy was noted. Patients preferred DOACs but expressed concern regarding the difference in monitoring between warfarin and DOACs. However, they resorted to trusting the professionals knowledge and desired access to dedicated anticoagulant services longterm. | This is a sub-study from a main study where VTE patients were also recruited. |
| Rush *et al.,* 2019 (50) | Mixed methods | 101 patients  15 physicians  Canada | To obtain patient and primary care physician perspectives of rural AF care and virtually delivered AF care being considered as an option | The most problematic emotional issue was anxiety about stroke and bleeding. Patients and physicians identified issues related to co-morbidities and limited opportunities for patient education | Study was mainly about general patient care rather than medication management but some reports on perspectives on medication, |
| Murphy, Kirby and Bradley, 2020 (51) | Quantitative | 309 GPs  Ireland | To examine general practitioner’s experience and practice of follow-up for managing AF patients prescribed DOACs | There was suboptimal monitoring of renal and hepatic impairment of older patients on DOACs | This study was done when DOACs were still relatively new. The situation may have changed now with more recent guidelines and the diffusion of DOACs into routine practice. |
